# Non-Invasive Point-of-Care Detection of Methamphetamine and Cocaine via Aptamer-Based Lateral Flow Test

**DOI:** 10.3390/bios15010031

**Published:** 2025-01-09

**Authors:** Bilge Erkocyigit, Ezgi Man, Ece Efecan, Ozge Ozufuklar, Deniz Devecioglu, Basak Bagci, Ebru Aldemir, Hakan Coskunol, Serap Evran, Emine Guler Celik

**Affiliations:** 1 Department of Biotechnology, Institute of Natural Sciences, Ege University, 35040 Izmir, Türkiye; 2 Department of Biochemistry, Faculty of Science, Ege University, 35040 Izmir, Türkiye; 3 Department of Psychiatry, Ataturk Educational and Research Hospital, Katip Celebi University, 35360 Izmir, Türkiye; 4 Department of Psychiatry, Faculty of Medicine, Izmir Tinaztepe University, 35400 Izmir, Türkiye; 5 Department of Psychiatry, Faculty of Medicine, Ege University, 35040 Izmir, Türkiye; 6 Department of Bioengineering, Faculty of Engineering, Ege University, 35040 Izmir, Türkiye; 7 Ege Science Pro Scientific Research Inc., 35040 Izmir, Türkiye

**Keywords:** lateral flow assay, aptamer, point-of-care, saliva drug test, abuse drug testing

## Abstract

Drug abuse is a major public problem in the workplace, traffic, and forensic issues, which requires a standardized test device to monitor on-site drug use. For field testing, the most important requirements are portability, sensitivity, non-invasiveness, and quick results. Motivated by this problem, a point of care (POC) test based on lateral flow assay (LFA) was developed for the detection of cocaine (COC) and methamphetamine (MET) in saliva which has been selected as the matrix for this study due to its rapid and non-invasive collection process. In the design strategy of an LFA test, the use of gold nanoparticles (AuNPs) with strong optical properties has been combined with the advantages of selecting aptamers under in vitro conditions, making it a highly specific and stable recognition probe for the detection of small molecules in saliva. The developed aptamer-based LFA in a competitive format, was able to detect COC and MET in synthetic saliva at concentrations as low as 5.0 ng/mL. After analytical performance studies, the test system also detected COC and MET in real patient samples, which was verified by chromatographic methods.

## 1. Introduction

Cocaine (COC) and methamphetamine (MET) are two illicit drugs that affect the central nervous system in different ways. COC is a local anesthetic that blocks the reuptake of the neurotransmitter’s norepinephrine, dopamine, and serotonin. This action leads to an increase in the levels of these neurotransmitters in the brain, which produces a euphoric effect. However, the long-term use of COC can cause damage to the central nervous system, leading to cognitive and motor dysfunction. MET, on the other hand, stimulates the release of and blocks the reuptake of these same neurotransmitters, producing a more intense and longer-lasting high. This stimulation of the central nervous system can cause a range of adverse effects, including anxiety, insomnia, and psychosis. Chronic use of MET can lead to neurotoxicity, which can result in long-lasting cognitive and motor deficits [1].

The United Nations World Drug Report 2022 has revealed concerning trends regarding MET and COC use. The report indicates that there has been a significant increase in the number of people using these drugs in recent years. In 2020 alone, 34 million people used MET, while 21.5 million people used COC. These updates are alarming, especially when considering that MET use now accounts for 0.7% of the global population, while COC use accounts for 0.4%. The report highlights the need for improved analytics to strengthen early warning and evidence-based intervention strategies [2].

The detection of illicit substance use is crucial to ensure safety in various settings. Saliva is one of the most important alternative matrices for the rapid detection of such drugs. Saliva testing has several advantages including non-invasive method of testing and ease of collection. This provides for collecting samples under direct observation at the workplace and in traffic, which is not always possible using urine samples. Collecting samples under direct observation helps to avoid the problems encountered when collecting urine samples, such as misleading results [3,4]. The transfer of substances from plasma to saliva is known to occur through passive diffusion. Passive diffusion is partially dependent on the size of the molecule, allowing it to pass through the saliva. COC and MET can be detected in saliva samples, even if they are taken invasively [3,5]. This suggests that saliva can be considered a suitable matrix for point-of-care (POC) diagnostics [6,7]. For cases in which drugs are not taken orally, substances such as COC and METs can pass from the bloodstream into saliva by passive diffusion. This process depends on the physicochemical properties of the drug, such as molecular weight and lipophilicity, which enable it to cross cell membranes and reach the salivary glands. When COC or METs are not taken orally, their presence in saliva may be at lower concentrations compared to direct oral exposure. However, their metabolites, such as benzoylecgonine for POP and amphetamine for MET, can still be detected in saliva due to systemic circulation and elimination pathways. In non-oral routes of administration, such as intravenous or inhalation, drug concentrations in saliva typically reflect plasma levels, but may be lower than concentrations in urine due to reduced drug accumulation in the oral cavities. Despite these challenges, saliva testing remains effective in detecting recent drug use as it provides a snapshot of free drugs and metabolites circulating in the body regardless of the route of administration [8,9,10]. The metabolic activity of COC and MET plays an important role in their detectability in biological matrices. Cocaine undergoes rapid hydrolysis and is metabolized primarily to benzoylecgonine and ecgonine methyl ester [10,11,12,13]. The major metabolite benzoylecgonine has a longer half-life and is commonly detected in saliva as an indicator of cocaine use. Methamphetamine is metabolized by oxidative and enzymatic pathways to amphetamine and other minor metabolites [13,14,15]. The presence of these metabolites in saliva highlights their systemic circulation and offers an extended window of detection even when the parent drug is no longer present. However, the levels of metabolites can vary depending on individual metabolic rates, route of administration, and time since ingestion, and require robust biosensing systems to discriminate between parent compounds and metabolites [16] Hence, saliva is considered to be the ideal and non-invasive matrix to evaluate whether a person is under the influence of substances for the workplace and roadside drug testing.

Detecting illegal substances such as COC and MET has traditionally relied on laboratory-based methods such as gas chromatography-mass spectrometry (GC-MS) and liquid chromatography-mass spectrometry (LC-MS) [3,5]. While these techniques provide great sensitivity and specificity, they are resource-intensive, time-consuming, and need trained staff, limiting their application in POC setting. Immunoassays, such as enzyme-linked immunosorbent assays (ELISAs), are also widely employed; however, they present with issues such as antibody stability, cost, being time-consuming and susceptible to environmental factors [17]. These limitations highlight the need for quick, affordable, and non-invasive solutions.

Electrochemical detection techniques have also gained popularity due to their great sensitivity, specificity, and applicability to a variety of analytes, including COC and MET. These methods often use electrodes modified with nanomaterials or aptamers to improve signal transmission and detection limitations. For example, carbon-based electrodes and metal nanoparticles have been used in electrochemical biosensors to detect COC and MET in biological fluids [18,19]. These platforms provide quantitative analysis, are portable, and may be linked to point-of-care systems. The combination of electrochemical detection with aptamer-based platforms expands the adaptability and utility of biosensors in forensic and clinical diagnostics.

Diagnostic devices called lateral flow assays (LFAs) can provide rapid POC results. One of the most significant benefits of LFAs is their high speed and cost. The results of these tests can be obtained within minutes, making them ideal for rapid diagnosis. Compared to laboratory tests, LFAs are considerably less expensive and user-friendly, particularly for the resource limited settings [6,20]. Sandwich testing and competitive testing are the two main formats used by LFAs. The sandwich format is used to detect larger molecules such as proteins that can be captured by more than one antibody [7,21]. On the other hand, the competitive format is used to detect smaller molecules such as illicit substances, toxins, drugs, and hormones [22,23,24,25]. In this format, the sample competes with a labeled antigen for binding to the capture antibody. The more analyte present in the sample, the less labeled antigen binds to the capture antibody. Analyte concentration in the sample is inversely proportional to the signal produced [7,21].

In conventional LFAs, antibodies are commonly employed as the capture molecules due to their specific binding capabilities toward target antigens. However, antibodies are susceptible to pH and temperature variations, leading to their instability and aggregation, thus compromising their overall performance and reliability. Moreover, antibody production involves time-consuming and expensive processes, which restrict their utility in certain applications. To overcome these limitations, aptamers have emerged as a promising substitute for antibodies in LFAs. Aptamers are short single-stranded DNA or RNA molecules that can selectively bind to a wide range of targets, including proteins, small molecules, and even whole cells, with high affinity and specificity. They are generated through a process called Systematic Evolution of Ligands by Exponential Enrichment (SELEX), where a large pool of random oligonucleotides is iteratively enriched for sequences that exhibit binding affinity towards the desired target [26]. Aptamers offer several advantages over traditional biomolecules like antibodies, including superior stability, reproducibility, and facile chemical synthesis. Their small size, lack of immunogenicity, and ability to be easily modified with various labels and functional groups make them valuable tools in diverse fields such as diagnostics, therapeutics, and biosensing [17,27]. Moreover, aptamers can be rapidly developed against a wide range of targets, enabling the swift design and implementation of aptamer-based assays [28,29]. Aptamer-based LFAs have demonstrated versatility in a wide variety of applications. An important application of LFAs based on aptamers is the determination of ions in water, which is crucial to maintaining the safety of drinking water. Furthermore, these assays can also be used to detect toxins and pesticides in foods, which can help ensure the safety of the food we consume [30,31,32,33,34,35,36,37]. Another important application of aptamer-based LFAs in the field of medicine is to identify biomarkers for early and rapid detection of diseases. This can be particularly useful for diseases that are difficult to diagnose using traditional methods [38,39,40,41,42,43]. Using aptamer-based LFAs for the detection of exosomes from human lung cancer cells, Yu and colleagues demonstrated the potential of this technology in cancer diagnosis [44]. Aptamers have also been used in electrical and colorimetric studies to identify COC and MET [18,45,46,47,48].

Recent advances in biosensing technologies, particularly LFAs, have revolutionized in situ detection capabilities for use in resource-limited environments, workplaces, and traffic checkpoints [20]. Inherent limitations of antibodies in traditional LFAs, such as instability and high production costs, have led to the search for aptamer-based alternatives. Since aptamers offer superior stability, specificity, and ease of chemical modification, they can target a wide range of analytes, including small molecules such as COC and MET [27,28]. The integration of aptamers with gold nanoparticles (AuNPs) further enhances the performance of LFAs. Aptamer-gold nanoparticle (Apt-AuNP) conjugates exhibit strong optical properties, stability under variable conditions, and precise target recognition. In competitive LFA formats, these conjugates compete with labeled antigens for binding sites, enabling the detection of low-molecular-weight analytes and providing high sensitivity and specificity [45]. Despite these advances, few studies have investigated aptamer-based LFAs for detecting illicit substances in noninvasive matrices such as saliva.

This study focuses on the rapid detection of COC and MET using an aptamer-based LFA system. Apt-AuNP conjugates were used as labeling agents, enabling rapid and easy determination of target drug molecules at the nanogram level. Both synthetic and real saliva samples were tested, and the system successfully detected the presence of COC and MET in both types of samples. The sensitivity and specificity of the test system make it a promising tool for rapid and reliable detection of illicit substances in saliva.

## 2. Materials and Methods

### 2.1. Reagents and Materials

COC and MET standard solutions were purchased from Cerilliant. Gold nanoparticles (AuNP) were obtained from Cytodiagnostics. Bovine serum albumin (BSA), sodium chloride (NaCl), sodium azide, Tween 20, tris(2-carboxyethyl)phosphine (TCEP), 6-mercapto-1-hexanol (MCH), and avidin, were purchased from Sigma-Aldrich (St. Louis, MO, USA). Saliva collection swabs and storage tubes were purchased from Salimetrics. The LFA kit prototype has 3D printed by MK PROJE Software and Engineering Services Inc. (Izmir, Türkiye).

Particle size measurement device (Dynamic Light Scattering (DLS), Malvern Nano ZS, Malvern, UK), NanoDrop UV-Visible Spectrophotometer (Thermo Fisher Scientific, Waltham, MA, USA) were used for the characterization of the conjugates. A dispenser (IsoFlow Dispenser, Imagene Technology, Lebanon, NH, USA) was used to design the lateral flow assays.

### 2.2. Detection Principle and 3D Prototype of Aptamer-Based Lateral Flow Assay

**Scheme 1:**
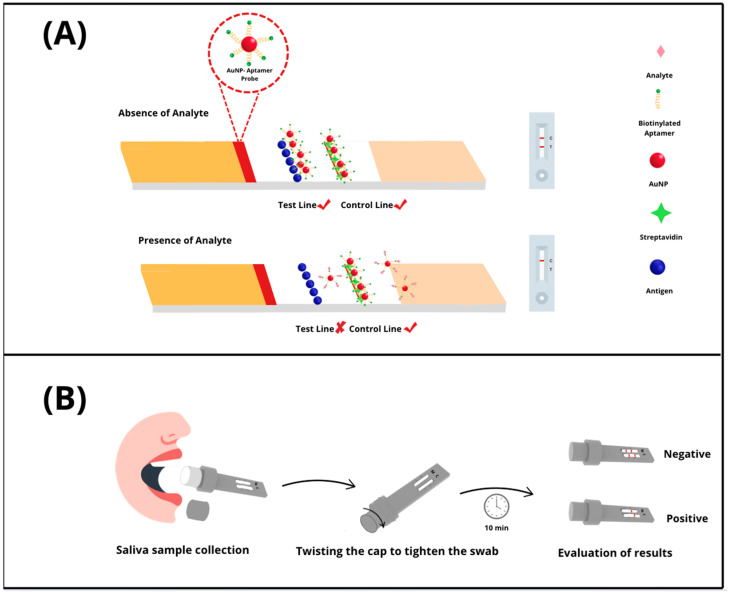
(**A**) Principle of aptamer-based LFA. (**B**) 3D Prototype of aptamer-based LFA.

Based on the principle of competitive testing, the test system was designed to detect low-molecular-weight analytes, COC and MET. In this format, the antigen-BSA conjugate on the T-line and analyte in the sample compete for binding to the Apt-AuNP probe were prepared as detector reagents. When samples without COC or MET analytes were applied to the strip, the Apt-AuNP probe in the conjugate pad was released. The probe moving across the NC membrane was captured by immobilized antigens as it passed over the T line. Biotin-terminated aptamers on the uncaptured probes were captured by streptavidin immobilized on the C line, resulting in the formation of red bands in both T and C lines on NC membrane. However, if the sample contains COC or MET, the analyte in the sample binds to the aptamer on the Apt-AuNP probe and prevents the binding of the probe to the antigens immobilized on the T line. In this case, no signal was generated on the T line, and the biotin end of the aptamer was captured by streptavidin as the conjugate passed over the C line, resulting in a red signal only on the C line. In this test format, a single line on the C line was considered positive, as shown in Scheme 1A. A model prototype of the test kit was designed by 3D printing. In this one-step rapid diagnostic kit prototype, after collecting the saliva sample, a mechanical effect was created by utilizing the cap attached to the swab. This action facilitated the movement of the saliva sample along the test kit membranes. The test result was observed within 10 min, as shown in Scheme 1B.

### 2.3. Synthesis of Aptamers with GO-SELEX Method

Graphene oxide-assisted systematic evolution of ligands by exponential enrichment (GO-SELEX) method was used for the selection of aptamers [49,50]. During the GO-SELEX rounds, binding buffer (100 mM NaCl, 2 mM MgCl_2_, 2.7 mM KCl, 10 mM Na_2_HPO_4_, 1.8 mM KH_2_HPO_4_, pH 7.0) in which target molecules were incubated with the DNA library was prepared and filtered through a 0.2 μm sterile filter [51]. Alpha Library was used as a starter DNA pool for in vitro selection. The reverse primer sequence was modified with phosphate groups for Lambda exonuclease digestion to obtain ssDNA [52]. The library was denatured at 95 °C for 5 min and cooled on ice for 5 min and incubated 25 °C before GO-SELEX step for the best conformation.

Next, 1.0 nmol of the ssDNA library was used in the first round of SELEX. The ssDNA library was incubated with 25 mg of graphene oxide (GO) at 25 °C for 1 h with shaking at 1000 rpm, and the ssDNA molecules were adsorbed onto the GO surface via π–π stacking [53]. Unbound ssDNA molecules were removed by washing with the binding buffer three times. The ssDNA molecules adsorbed on GO were obtained by centrifugation (15,000 rpm, 15 min, 25 °C), and the precipitate was resuspended in 200 μL binding buffer. The target molecules methamphetamine (5 nmol) and cocaine (5.0 nmol) were added to the medium, and incubation was continued at 25 °C for 2.5 h with shaking at 900 rpm. Following incubation, the mixture was centrifuged at 15,000 rpm, 15 min, and 25 °C. The supernatant containing the desorbed ssDNA sequences was collected then the recovered library was amplified by polymerase chain reaction (PCR) [54]. Initial denaturation was performed at 95 °C for 3 min, followed by each cycle of 95 °C denaturation for 30 s, 64 °C annealing for 30 s, and 72 °C extension for 30 s. The final extension was performed at 72 °C for 2 min.

The double-stranded DNA (dsDNA) product was purified by using PCR clean-up kit (Machery-Nagel, Düren, Germany), and then purified by Lambda exonuclease digestion [52]. The ssDNA pool was used in the next SELEX round. As given in Tables S2 and S3, incubation time, as well as the amount of methamphetamine, cocaine, and benzoylecgonine was reduced to increase the stringency of SELEX. In order to achieve the selectivity against the target molecule, counter-SELEX was performed in the 7th round of SELEX with codeine. The final ssDNA pool obtained from the 7th round was cloned by using the InsTAClone PCR cloning kit (ThermoFisher Scientific, Waltham, MA, USA). Then, 40 of the randomly selected clones were sequenced by SenteBiolab (Ankara, Türkiye) for each selection. The sequences were analyzed by using the MEME suite [55] (Figure S1). Online mfold web server [56] was used for prediction of the enriched secondary motifs (Figure S2). Selected aptamer candidates were synthetized by chemically from SenteBiolab (Ankara, Türkiye) (Table S1).

### 2.4. Optimization Studies for Apt-AuNP Probe

To select the most appropriate Apt-AuNP probe, AuNPs of different sizes (40 nm and 20 nm) and surface stabilizations (citrate-stabilized and surfactant-stabilized) were evaluated. In conjugates prepared with 40 nm and 20 nm standard AuNPs, a color change from red to purple was observed upon salt addition, as shown in Figure S5. This color shift, as reported in previous studies, is attributed to aggregation [38]. When AuNPs lose their stabilized form, they interact with each other, forming larger structures that eventually collapse into aggregates. Aggregation is one of the most important parameters that prevent signal formation in LFA tests. Therefore, the preparation of the most suitable non-aggregated AuNP conjugate is an important optimization step. The conjugate prepared with 40 nm stabilized AuNP (surfactant stabilized) was more stable against salt addition. In many studies on aptamers, the salt-aging method has been used to bind more aptamers to the AuNP surface [41]. Based on these findings, 40 nm surfactant-stabilized AuNPs were chosen as the most suitable for further experiments.

To prevent non-specific interactions in the test strips, blocking of Apt-AuNP conjugates was performed with BSA and MCH at different ratios [43,57], and the results were compared with unblocked conjugates. As shown in Figure S6, aggregation occurred in conjugates blocked with MCH. For the unblocked conjugate, applying a negative sample resulted in line formation only at the control line, with no test line visible (Figure S7A). In the BSA-blocked conjugate, as a result of negative sample application, a line was formed in the test line and its visibility decreased after a short time (Figure S7B). Based on these results, subsequent conjugates were blocked with 10 mg/mL BSA.

To determine the optimum aptamer concentration, three different conjugates were prepared with the final aptamer concentrations of 5.0, 10, and 25 µM, and their applicability on the test strip was evaluated. In the test strip where the aptamer concentration was 10 µM for COC and 5.0 µM for MET, the signals on the test and control lines remained stable following the application of a negative sample. When a positive sample was applied, as expected in the competitive tests, no signal was observed in the test line and only the control line was observed (Figures S8 and S9).

The synthesized aptamers were designed with an -SH group at one end for conjugation via gold–thiol interactions and a biotin group at the other end for interaction with streptavidin on the control line. To evaluate whether the biotin modification affects the aptamer’s ability to recognize the target substance and influence the test response, conjugates were prepared using both biotin-ended aptamers and aptamers without biotin. As shown in Figure S10, both negative and positive responses were observed for MET and COC strips when biotin-ended aptamers were used.

In order to determine the optimum salt concentration in the salt aging method, a conjugate was formed by adding NaCl with the final concentrations of 30 µM, 70 µM, and 100 µM and tested on the test strips. As shown in Figures S11 and S12, increasing the concentration of added NaCl resulted in enhanced signal generation. Based on this, we can say that more aptamers are loaded on AuNP with increasing salt concentration [33]. In the following studies, the salt concentration was chosen as 100 µM.

### 2.5. Optimization of LFA Strip Preparation

First, the blocking conditions were evaluated for the nitrocellulose membrane. The test and control lines on the membrane treated with the blocking solution were not formed after the application of either negative or positive samples (Figure S13A). In contrast, on the unblocked membrane, the flow of the conjugate after release from the conjugate pad was consistent with that observed in standard strips, and the membrane successfully responded to both negative and positive samples (Figure S13B). Therefore, the unblocked nitrocellulose membrane was selected for further experiments.

The amount of conjugate applied to the conjugate pad was evaluated as another parameter which is important for the consistent release, efficient flow, and reliable signal formation in the analytical membrane. Conjugates were applied to the conjugate pad at different dilutions with a total volume of 75 µL and the membrane was cut into 5 cm length and 0.5 cm width. (The dilution amounts were given in supplementary information). In the undiluted conjugate applied membrane, it failed to move across the membrane due to an excessive amount of conjugates. Consequently, it could not reach the control line (Figure S14A). In the strip shown in Figure S14B, the conjugate moved along the membrane, but after the signal temporarily appeared on the test line, while an intense signal was observed on the control line. As shown in Figure S14C,D, the signal intensity on the test and control lines increased in parallel to the decreased dilution ratios. This can be related to the surfactants in the conjugate-blocking solution. The flow rate on the membrane was high due to the surfactants in the conjugate blocking solution, and low signal intensity was observed due to the increased migration rate in the test line [30]. Based on these results, 1:1 dilution was found to be suitable for further experiments.

In order to determine the optimal concentration of antigen-BSA for the test line, 1.0 mg/mL and 2.0 mg/mL concentrations were applied. As shown in Figure S15A, the test and control lines provided weak signal when a negative sample was used. In contrast, as shown in Figure S15B, increasing the antigen concentration on the test line resulted in stronger signals on both the test and control lines. In both conditions, the signal intensity of the test line was lower than that of the control line. This difference is attributed to the behavior of the aptamer-AuNP conjugate prepared by the adsorption–desorption method. Initially, the conjugate binds to the test line; however, upon encountering the analyte-BSA, the aptamers detach from the AuNP and are retained at the control line, leading to a darker signal at the control line [38].

The drying conditions of conjugate pads in an air-conditioned cabinet was another optimization parameter. The strip prepared with the conjugate pad dried at 20% humidity for 2 h showed a delayed release of the conjugate from the pad and a low signal, as shown in Figure S16A. The strip prepared with the conjugate pad dried at 37 °C for 2 h under humidity-free conditions did not fully release the conjugate and left as a dark stain on the nitrocellulose membrane. In the strip prepared with the membrane dried for 30 min under moisture-free conditions, the control and test lines were observed more clearly. As a result, the drying conditions were selected as 37 °C for 30 min without moisture.

To test the effect of the sample application pad, strips were prepared using three types of sample pads. As shown in Figure S17A, while the negative sample was applied to the strip prepared with Grade 238 cotton fiber, no conjugate was released from the conjugate pad. When the negative sample was tested on the strip prepared with Grade 601 sample application pad, a very small amount of conjugate was released, and very weak test and control lines were formed. The effect of using a glass fiber pad on the working performance of the strip was as shown in Figure S17. As a result, clearer control and test lines were obtained.

### 2.6. Preparation of Aptamer-AuNP Conjugate

Aptamer-AuNP conjugate was prepared by modifying the covalent binding method based on the gold–sulfur reaction [41,42]. Firstly, 100 µM thiol-terminated aptamer was added to the folding buffer (1.0 mM MgCl_2_, 1× PBS 7.4). The solution was heated at 90 °C for 5 min. It was then allowed to cool at room temperature for 15 min. To activate the aptamer, 20 mM TCEP was added and incubated at room temperature for 1 h. On the other hand, 4.0 mL AuNP solution was centrifuged at 4 °C, 4500 rpm for 20 min and the supernatant was discarded. Then, 5.0 µL of activated aptamer was added to 100 μL AuNP and was incubated overnight in the dark for at least 8 h. The salt aging method was applied to bind more aptamer to the AuNP surface. For this purpose, 1.0 M NaCl was added to the solution in 0.5 µL drops and incubated at 4 °C for 24 h. At the end of incubation, centrifugation was performed at 4500 rpm for 20 min. Pellets were washed 3 times with ultrapure water. Then, 10 mg/mL BSA (bovine serum albumin) was added to block the empty spaces on AuNP surfaces and incubated for 30 min. The mixture was centrifuged at 4500 rpm for 10 min and washed 3 times with ultrapure water. Finally, the pellets were resuspended with conjugate blocking solution (sucrose, BSA, NaCl, sodium azide, Tween 20 in pH 7.4 phosphate buffer).

### 2.7. Preparation of the Lateral Flow Strips

The LFA strip is composed of several layers, including a backing card, sample pad, conjugate pad, nitrocellulose membrane (NC), and absorbent pad. The sample pad, which was made of glass fiber material, was first blocked with a solution containing Tween 20, sucrose, dextran, sodium azide, and ultrapure water and it was then dried overnight. The blocking solution is an essential component of the LFA strip, as it helps to prevent non-specific binding of the target analyte. By blocking the sample pad, any possible interfering substances in the sample that could cause false positive results in the test are prevented from binding to the pad [29]. In order to prepare the NC membranes for the detection of both COC and MET, firstly, a dispenser was used to draw 2.0 mg/mL avidin on the control (C) line of the NC membrane with the pore size of 10 nm. Next, for the COC strip, 2.0 mg/mL COC-BSA was drawn on the test line, whereas for the MET strip, 2.0 mg/mL MET-BSA was drawn on the test line, and the prepared NC membranes were then left to dry in a chamber for 4 h at 37 °C. Then the Apt-AuNP conjugate was dispensed into the conjugate pad and allowing it to dry for 2 h at 37 °C. Finally, all the components were carefully placed on the backing card and approximately 0.2 cm thick strips were accurately cut using a pair of scissors.

### 2.8. Preclinical Studies

Ethical approval to conduct preclinical studies was obtained from the Izmir Katip Celebi University, Clinical Research Ethics Committee with decision number 7, dated 6 January 2022. For this purpose, 40 saliva samples were collected from volunteers who signed an informed consent form in Izmir Tinaztepe University Private Buca Hospital and Izmir Ataturk Training and Research Hospital. The samples were collected using oral swabs and were stored at −20 °C. After the samples were analyzed using the developed test system, they were applied for confirmation by chromatographic testing.

## 3. Results and Discussion

### 3.1. Characterization Studies for Binding Affinity of the Selected Aptamers

Isothermal titration calorimetry (ITC) is a label-free calorimetric technique to study aptamer-target interactions [58]. ITC is a gold standard method that allows the determination of thermodynamic parameters of biomolecular interactions, including affinity (KA), enthalpy (ΔH), entropy (ΔS), and stoichiometry (n) [59]. Binding reactions that are energetically favorable exhibit negative values of free energy, ΔG = RTlnKD. The mathematical expressions for the relationships between ΔG, ΔH, and ΔS are as follows: ΔG = ΔH-TΔS [60]. ITC experiments were performed with PEAQ-ITC (Malvern Panalytical, Malvern, UK) at room temperature. Before measurement, the samples were degassed for 5 min. Binding buffer was composed of 100 mM NaCl, 2 mM MgCl_2_, 2.7 mM KCl, 10 mM Na_2_HPO_4_, 1.8 mM KH_2_HPO_4_, (pH 7.0). Aptamers were folded at 95 °C for 3 min, cooled at 4 °C and then incubated at 25 °C before the measurements. ITC experiments were completed with 19 injections. The sample cell contained 300 μL of aptamer and the syringe contained 40 μL of target molecule. Then, 250 μM methamphetamine was titrated into 20 μM methamphetamine-aptamer. Next, 450 μM cocaine was titrated into 25 μM cocaine-aptamer (Figures S3 and S4). Titration data were analyzed with PEAQ-ITC software (Version 1.2). Dissociation constant (Kd), as well as the thermodynamic parameters including enthalpy change (ΔH) and Gibbs free energy change (ΔG) were calculated [61,62] (Table S4).

The sequence analysis was completed after in vitro aptamer selection rounds. The consensus motif enrichment results from MEME suit analysis and the *p* values were used to choose the aptamer candidates (Figure S1). Figure S2 shows the predicted mfold secondary structures of aptamers. Isothermal titration calorimetry (ITC) was used to measure the binding affinity of cocaine and methamphetamine aptamers [63]. The binding affinities of COC and MET aptamers were determined as 306 nM and 570 nM, respectively (Figures S3 and S4, and Table S4).

### 3.2. Characterization of Aptamer-AuNP Conjugate

A conjugate was prepared by coating AuNPs with biotin-ended aptamers by gold-thiol interaction. The conjugate was used as a recognition probe in this test system. The characterization of the conjugates was performed by scanning electron microscopy (SEM), dynamic light scattering spectrometry (DLS), and UV-Visible Nanodrop devices to determine whether the aptamers were coated on AuNPs.

**Figure 1:**
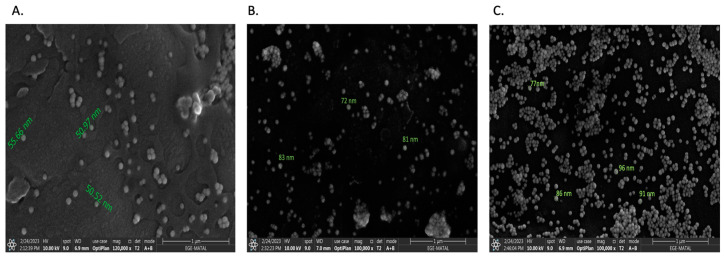
SEM images of (**A**) AuNP, (**B**) COC-AuNP, and (**C**) MET-AuNP.

The size of the AuNPs before conjugation was approximately 58.7 nm. After COC and MET modifications, a size distribution of 94.92 nm and 95.87 nm was measured. Based on the measurement results, it is considered that the size increases with aptamer binding to the surface of the AuNPs. The zeta potential of plain AuNPs was measured as −36.77 mV (Table 1). With the binding of COC and MET aptamers to the AuNP surface, a positive change in the zeta potential was observed. As a result of the measurements, it was interpreted that the binding of aptamers to the surface of AuNP shifted surface charge to the positive direction. Particle size and zeta potential measurements confirmed that the aptamer binding to the AuNP surface was successfully performed. The SEM images (Figure 1) confirm the data obtained from the DLS results. The observed increase in particle size confirms successful conjugation, as indicated by the binding of the aptamer to the AuNP. The sizes measured by DLS were slightly higher than those observed in the SEM images. This discrepancy could be attributed to the broader size distribution in the sample, including the presence of very large particles, which may influence the overall diameter calculated through light scattering, thereby increasing the average error. Additionally, the presence of particle aggregates in the sample might also contribute to the observed mismatch in size measurements [64].

**Figure 2:**
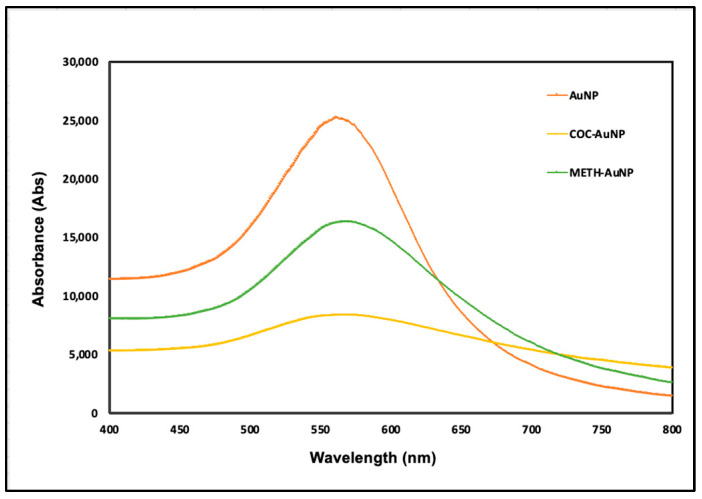
Spectrophotometric characterization with Nanodrop UV-Visible spectrometer.

Since AuNPs have a maximum absorbance peak between 540 and 570 nm wavelength, the binding of COC and MET aptamers to the AuNP surface resulted in a decrease in the maximum absorbance peak (Figure 2). The decrease in the peak level confirmed the successful binding of the aptamers to the surface of the AuNPs.

### 3.3. Analytical Performance Studies

#### 3.3.1. Sensitivity and Specificity

For the determination of the linear range, COC standards added to synthetic saliva at concentration ranges of 1.0 µg/mL, 500 ng/mL, 100 ng/mL, 25 ng/mL, 10 ng/mL, 5.0 ng/mL were applied to the COC test strips. For the MET test strip, MET standards were added to synthetic saliva at concentrations of 200 ng/mL, 100 ng/mL, 50 ng/mL, 25 ng/mL, 10 ng/mL, 5.0 ng/mL were applied.

**Figure 3:**
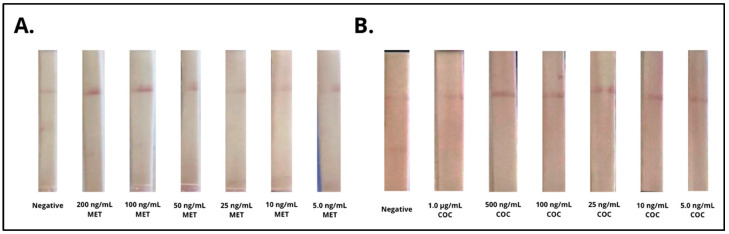
Determination of detection ranges for (**A**) MET strips and (**B**) COC Strips.

In the literature, the range of detectable concentrations of COC in saliva after cigarette ingestion was reported as 15.85–504.88 ng/mL as well as the range of detection in saliva after intravenous ingestion was 428–1927 ng/mL [5]. Positive responses were obtained from 1.0 µg/mL concentration to 5.0 ng/mL when COC standard solutions were applied. The oral fluid cut-off values for COC determined by organizations such as SAMHSA, DRUID, and Talloires are 8.0 ng/mL, 10 ng/mL and 10 ng/mL, respectively [13]. As seen in Figure 3, the cut-off value is 5.0 ng/mL in the strips we prepared. In a study conducted on the amounts of MET in saliva, MET concentrations varying according to dose intake and residence time in saliva were generally between 2.5–200 ng/mL [3]. Strips prepared for MET showed positive responses between 5.0 ng/mL and 200 ng/mL. Oral fluid cut-off values for MET determined by organizations such as SAMHSA, DRUID, and Talloires are 50 ng/mL, 25 ng/mL, and 20 ng/mL, respectively [13]. The cut-off value for the prepared MET strip was determined as 5.0 ng/mL. The results are shown in Figure 3A,B. Test responses clearly formed within 10 min and no false positives or false negatives were observed up to an hour.

**Figure 4:**
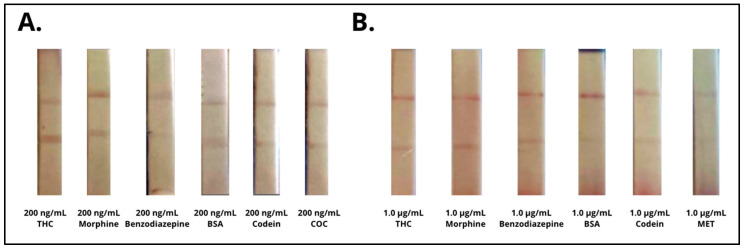
Interferences and cross-reactivity results (**A**) MET strips; (**B**) COC strips.

To evaluate the interference effect of other substances, tetrahydrocannabinol (THC), morphine and codeine as opioids, benzodiazepine as a sedative drug, and BSA as a large, structured protein were tested using COC and MET test strips. To verify the potential cross-interference effect, MET standard was applied to the COC test strip, and the COC standard was tested on the MET test strip. For MET, 200 ng/mL, the highest value within the detection range identified during the concentration experiment, was selected as the interferent concentration for this step. Similarly, for the COC experiments, interferents were tested at a concentration of 1.0 µg/mL for the same reason. As shown in Figure 4A,B, there was no response to THC, bezodiazepine, morphine, codeine, or BSA tested to detect the interference effect. In addition, no cross-reactivity was shown between the COC and MET.

The % sensitivity and % specificity values were calculated based on the number of true positive (TP) and true negative (TN) responding test strips for the total number of negative and positive COC and MET test strips tested repeatedly at different time intervals. Sensitivity and specificity values for COC and MET test kits were calculated according to the TP to P ratio and TN to N ratio, respectively [65]. The results are shared in Table 2.

#### 3.3.2. Storage Stability

**Figure 5:**
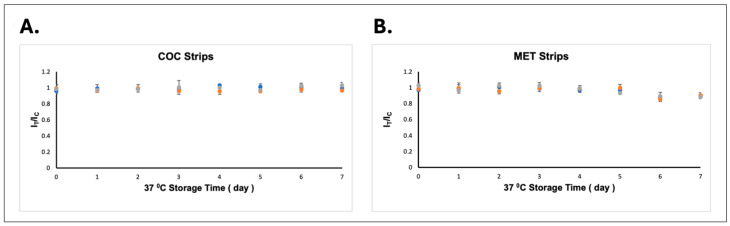
Accelerated aging test (**A**) COC strips and (**B**) MET strips.

To evaluate the storage stability of the developed test kits, an accelerated aging test was applied, and the results were captured from three positive and three negative tests per each day. In order to monitor stability, I_T_/I_C_ ratios (I_T_: Color intensity of T line, I_C_: Color intensity of C line) were calculated according to negative tests by using ImageJ software (Version 1.53k) (Figure 5). According to the applied reference method, 37 °C for 7 days was equivalent to one year at room temperature [66]. Moreover, one day of incubation at 37 °C represents 1.5 to 2 months of storage at room temperature [67]. The COC test strips responded to negative and positive tests after seven days of incubation. It can be interpreted that the storage stability of COC strips is equivalent to approximately one year.

On the MET test strip, a decrease in negative responses was observed on days 6 and 7. Considering that one day corresponds to a time period of approximately 1.5–2 months, it is concluded that there is a storage stability of approximately 10 months for MET strips.

### 3.4. Preclinical Study and Chromatographic Validation

Saliva samples were collected using oral swabs from a total of 40 volunteers, including both substance users and non-users, and stored at −20 °C until further testing. The samples were then thawed at room temperature and tested on the prepared test strips. Finally, validation studies were performed by liquid chromatography quadrupole time-of-flight mass spectrometry (LC-QTOF/MS) to verify the results obtained using the designed test kit.

**Figure 6:**
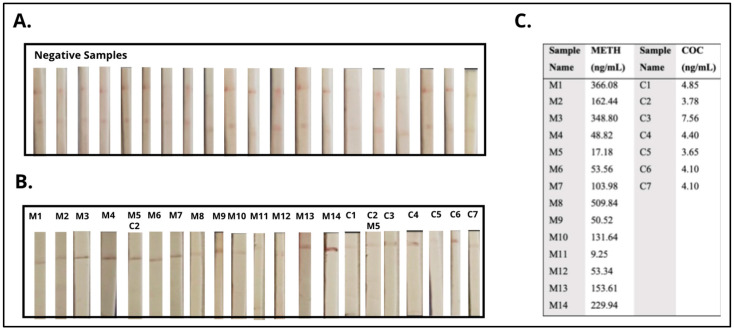
Application of real saliva samples: (**A**) negative samples; (**B**) samples containing COC and MET; (**C**) LC-QTOF/MS results of samples containing COC and MET.

Saliva samples obtained from MET users were numbered M1 to M14. Similarly, the samples for COC were numbered C1 to C7. Samples M5 and C2 contained COC and MET simultaneously. As shown in Figure 6A,B, the test kit provided true positive and true negative results for the applied 40 saliva samples. According to LC-QTOF/MS analysis results, calibration was performed in the range of 1.0–50 ng/mL for the COC samples, and the correct equation was found to be y = 1000000x + 775429, with an R2 value of 0.9988. For the MET samples, calibration was performed in the range of 1.0–100 ng/mL and the correct equation was y = 301459x + 549342, with an R2 value of 0.9978. The analyte concentrations of the samples are listed in Figure 6C. Chromatography results confirmed the presence of COC and MET in the positive saliva samples.

### 3.5. Prototype Design for Test Kit

**Figure 7:**
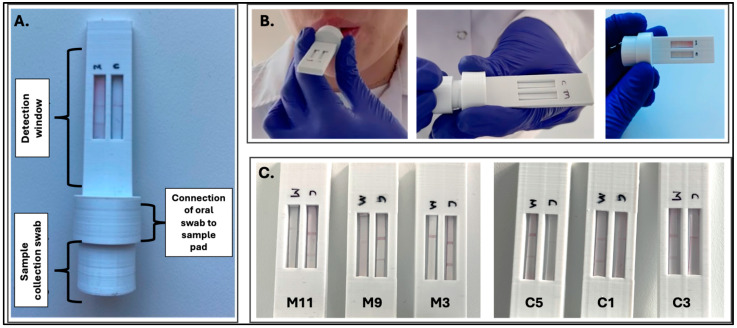
(**A**) Test kit prototype. (**B**) Sample collection and application process (**C**). Test results of positive samples by test prototype.

A prototype was designed using a 3D printer to facilitate sample collection in the field, enabling on-site applications such as traffic and workplace drug testing, while ensuring it is optimized as a rapid test candidate (Figure 7A). The test kit was designed for end users, providing a non-invasive and user-friendly application (Figure 7B). It is possible to detect both COC and MET simultaneously owing to the double observation window. In the prototype, saliva can be collected directly without additional swab application using a cotton in a round chamber which is connected to the test kit. (See Video S1 of the supporting information for the usage instructions of the designed test kit.). In the preclinical study, MET-positive samples M11 (9.25 ng/mL), M9 (50.52 ng/mL), and M3 (348.8 ng/mL) were applied to the prototype, representing low, medium, and high substance concentrations. For COC, samples C5 (3.65 ng/mL), C1 (4.85 ng/mL), and C3 (7.56 ng/mL) were applied as well (Figure 7C). With this application, the working performance of the test kit was demonstrated successfully.

## 4. Conclusions

This study successfully developed an aptamer-based LFA test kit for detecting COC and MET in saliva, providing a practical and efficient tool for POC applications in public safety and health screening. The use of aptamers as recognition elements highlights their potential as alternatives to antibodies, offering specificity and adaptability for detecting illicit substances. Although aptamer integration in LFA platforms is still emerging, this study demonstrates their feasibility for substance detection in saliva, addressing a critical gap in the field [68]. While line signal intensities in this study were sometimes weaker than those observed in antibody-based commercial LFA tests, the integration of aptamers represents a new frontier with opportunities for further improvement.

Overall, the findings of this study underscore the importance of further R&D efforts to refine signal quality and standardization. The demonstrated performance in preclinical studies highlights the test kit’s potential as a scalable, customizable, and user-friendly product for rapid substance detection. Designed for on-site testing, the kit ensures non-invasive, tamper-resistant sample collection, adaptable to emerging illicit substances, and testable in multiple biological fluids.

## Figures and Tables

**Table 1:** Size and zeta potential measurement results of AuNP, cocaine aptamer-AuNP conjugate (COC-AuNP), methamphetamine aptamer-AuNP conjugate (MET-AuNP).

Sample	Size (nm)	Zeta Potential (mV)
AuNP	58.7 ± 0.88	−36.77 ± 17.85
COC-AuNP	94.92 ± 12.65	−7.19 ± 7.81
MET-AuNP	95.87 ± 9.95	15.54 ± 6.63

**Table 2:** Sensitivity and specificity values for COC and MET strips.

	P	TP	N	NT	Sensitivity %	Specificity %
COC Strips	58	56	62	62	96%	100%
MET Strips	49	44	53	48	89%	90%

P = Total amount of positive sample-treated strips. TP = Amount of true positive response. N = Total amount of strips with negative sample applied. TN = Amount of true negative response.

## Data Availability

Data are contained within the article.

## Supplementary Materials

The following supporting information can be downloaded at: https://www.mdpi.com/article/10.3390/bios15010031/s1, Table S1: Aptamer Sequences of methamphetamine (MET) and cocaine (COC); Table S2: Summary of MET GO-SELEX conditions for Alpha library (see the text for details); Table S3: Summary of COC/benzoylecgonine GO-SELEX conditions for Alpha library (see the text for details); Figure S1: MEMEsuit analysis results (A) MEMEsuit results of COC/benzoylecgonine GO-SELEX, (B) MEMEsuit results of MET GO-SELEX; Figure S2: Mfold—predicted secondary structures of aptamers (A) COC, (B) MET; Figure S3: Molecular interactions of COC with COC aptamer mesured by ITC. 450 μM MET was titrated into 20 μM aptamer at 25 °C. Raw ITC data (top) and enthalpy change plotted against the COC:aptamer molar ratio (down); Table S4: ITC results of COC and MET aptamers; Figure S4; Molecular interactions of MET with MET aptamer measured by ITC. 250 μM MET was titrated into 20 μM aptamer at 25 °C. Raw ITC data (top) and enthalpy change plotted against the MET:aptamer molar ratio (down); Figure S5: Conjugates prepared with standard and stabilized gold nanoparticles (AuNPs); Figure S6: Conjugates blocked under different conditions. (1) Apt/AuNP ratio 1/20 2.0 µL 28 mM MCH 10 min (2) Apt/AuNP ratio 1/20 2.0 µL 14 mM MCH 10 min. (3) Apt/AuNP ratio 1/20 10 µL 10 mg/mL BSA 30 min. (4) Apt/AuNP ratio 1/20 Unblocked (5) Apt/AuNP ratio 1/10 5.0 µL 10 mM MCH 10 min. (6) Apt/AuNP ratio 1/10 5.0 µL 100 mM MCH 1 min; Figure S7: Strip prepared with (A) unblocked conjugate, (B) BSA blocked conjugate; Figure S8: COC strips with (A) 5.0 µM aptamer concentration (B) 10 µM aptamer concentration (C) 25 µM aptamer concentration; Figure S9: MET strips with (A) 5.0 µM aptamer concentration (B) 10 µM aptamer concentration (C) 25 µM aptamer concentration; Figure S10: (A) Strip prepared with aptamer without biotin for COC (B) Strip prepared with biotin-tipped aptamer for COC (C) Strip prepared with biotin-tipped aptamer for MET (D) Strip prepared with aptamer without biotin for MET; Figure S11: COC Strips (A) Strip prepared with conjugate with final concentration of 30 µM NaCl (B) 70 µM NaCl (C) 100 µM NaCl; Figure S12: MET Strips (A) Strip prepared with conjugate with final concentration of 30 µM NaCl (B) 70 µM NaCl (C)100 µM NaCl; Figure S13: (A) Nitrocellulose membrane with blocking solution (B) Nitrocellulose membrane without blocking solution; Figure S14: Negative sample applications on strips prepared by dilutions applied to conjugate. (A) 35 µL of undiluted conjugate was impregnated into the conjugate pad. (B) 15 µL conjugate was diluted with 60 µL conjugate blocking solution. (C) 25 µL conjugate was diluted with 50 µL conjugate blocking solution. (D) 37.5 µL conjugate was diluted with 37.5 µL conjugate blocking solution; Figure S15: (A) 1.0 mg/mL concentration antigen test strip (B) 2.0 mg/mL concentration antigen test strip; Figure S16: Strip formed by membrane dried at (A) 37 °C for 2 h under 20% moisture (B) 37 °C for 2 h under moisture-free conditions (C) 37 °C for 30 min under moisture-free conditions; Figure S17: Strip prepared with (A) Ahlstrom Grade 238 cotton fiber pad (B) Ahlstrom Grade 601 cotton fiber pad (C) Ahlstrom glass fiber pad.
