# Supplementary material for: Non-Invasive Point-of-Care Detection of Methamphetamine and Cocaine via Aptamer-Based Lateral Flow Test
Source: Biosensors (Basel). 2025 Jan 9;15(1):31. doi: 10.3390/bios15010031 (PMC11764150; doi:10.3390/bios15010031)
Supplement: Supplementary file 1 [file biosensors-15-00031-s001.zip › biosensors-3388213-supplementary.pdf]

Non-Invasive Point-of-Care Detection of Methamphetamine and Cocaine via Aptamer-Based Lateral Flow Test

Bilge Erkocyigit [1], Ezgi Man [2], Ece Efecan [1], Ozge Ozufuklar [1], Deniz Devecioglu [3], Basak Bagci [3], Ebru Aldemir [4], Hakan Coskunol [5], Serap Evran [2] and Emine Guler Celik [6,7,*]

[1] Department of Biotechnology, Institute of Natural Sciences, Ege University, 35040 Izmir, Türkiye
[2] Department of Biochemistry, Faculty of Science, Ege University, 35040 Izmir, Türkiye
[3] Department of Psychiatry, Ataturk Educational and Research Hospital, Katip Celebi University, 35360 Izmir, Türkiye
[4] Department of Psychiatry, Faculty of Medicine, Izmir Tinaztepe University, 35400 Izmir, Türkiye
[5] Department of Psychiatry, Faculty of Medicine, Ege University, 35040 Izmir, Türkiye
[6] Department of Bioengineering, Faculty of Engineering, Ege University, 35040 Izmir, Türkiye
[7] Ege Science Pro Scientific Research Inc., 35040 Izmir, Türkiye
* Correspondence: emine.guler.celik@ege.edu.tr

Table S1. Aptamer Sequences of methamphetamine (MET) and cocaine (COC).

Aptamer	Sequences (5'>3')
MET	AGGAATTCAGATCTCCCTGCAGAAAACAGACCACCCACTTTGAA[illegible]<br>CCACTTGACTCGAGGAGCTCAGGATCCCG
COC	AGGAATTCAGATCTCCCTGCAGTTCGAGGAAGAATAGACCTGCC[illegible]<br>TCTAATAG CTCGAGGAGCTCAGGATCCCG

Table S2. Summary of MET GO-SELEX conditions for Alpha library (see the text for details).

SELEX Round	MET concentration (pmol)	Codein concentration (pmol)	Incubation Time (min)
1	5000	-	150
2	3000	-	120
3	1000	-	100
4	750	-	60
5	500	-	60
6	100	-	60
7	-	500	45

Table S3. Summary of COC/benzoylecgonine GO-SELEX conditions for Alpha library (see the text for details).

SELEX Round	Cocaine / benzoylecgonine concentrations (pmol)	Codein concentration (pmol)	Incubation Time (min)
1	5000	-	150
2	2500	-	120
3	1000	-	100
4	500	-	60
5	300	-	60
6	200	500	60
7	100	-	30

A.

1.
***E*-value:** 4.4e+003 **Site Count:** 5 **Width:** 15

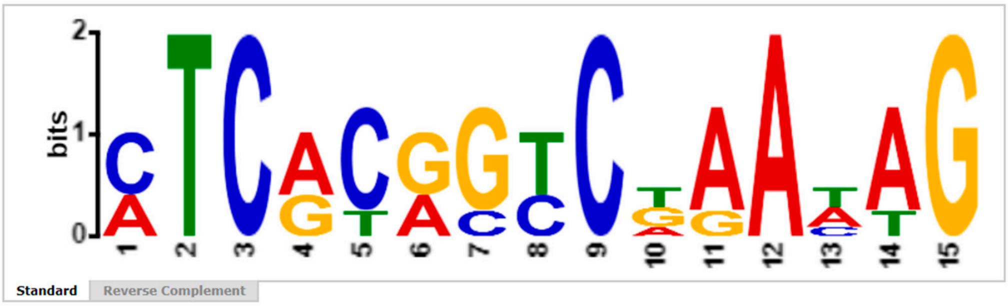

Log Likelihood Ratio: 70 **Information Content:** 20.2 **Relative Entropy:** 20.2 **Bayes Threshold:** 7.13858

Name	Strand	Start	*p*-value	Sites
4. 4	+	26	1.06e-8	ACCTGCCTGC CTCGCGGTCTAATAG
22. 22	-	2	1.70e-7	AGGTTTGAAC CTCACAGTCAAACAG G
8. 8	+	19	1.86e-7	CTTATAGCAC ATCATGGTCGAATAG CTGTAGC
11. 11	-	5	3.78e-7	CTAAAAAGGA ATCGCGCCCTAAAAG TACA
16. 16	-	7	7.65e-7	CGCCCATCAT CTCACAGCCGGAATG TGTTTT

B.

1.
***E*-value:** 2.4e+001 **Site Count:** 4 **Width:** 20

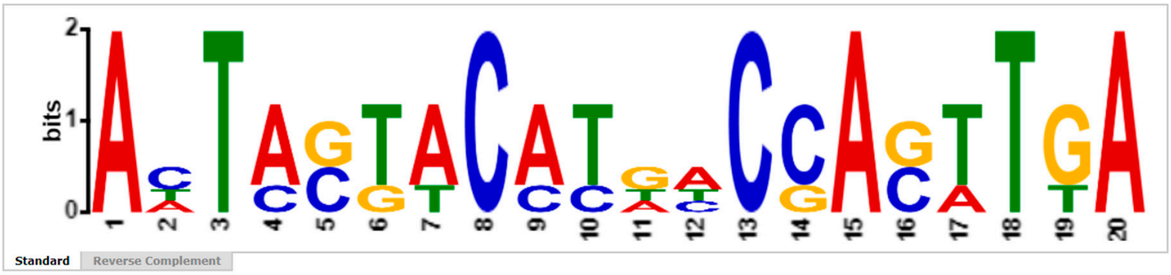

Log Likelihood Ratio: 77 **Information Content:** 27 **Relative Entropy:** 27.8 **Bayes Threshold:** 6.47573

Name	Strand	Start	*p*-value	Sites
4. 4	+	21	8.58e-12	ACCCACTTTG AATAGTACATGACCACTTGA
6. 6	-	1	2.62e-11	TGACGCATTG ACTAGTACATGACCACATGA
1. 1	+	25	6.64e-9	GACACTTCGC ATTCCGACATACCGAGTTGA
15. 15	-	5	7.30e-9	TTCAGCCGCT ACTACTTCCCTTCCAGTTTA ACGT

Figure S1. MEMEsuit analysis results A.) MEMEsuit results of COC/benzoylecgonine GO-SELEX, B.) MEMEsuit results of MET GO-SELEX

A. **B.**

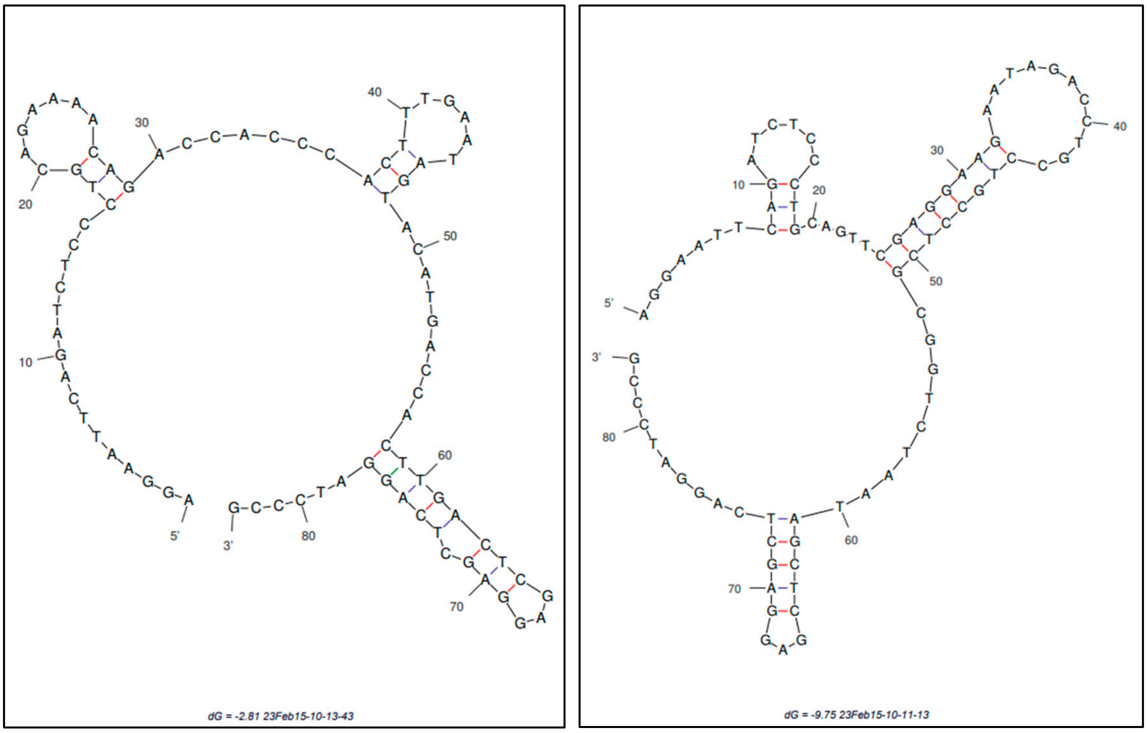

Figure S2. Mfold – predicted secondary structures of aptamers A.) COC, B.) MET

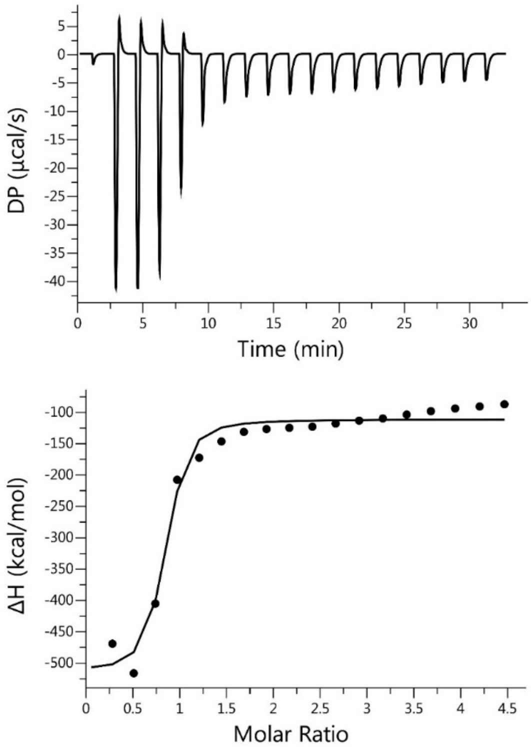

Figure S3. Molecular interactions of COC with COC aptamer mesured by ITC. 450 µM MET was titrated into 20 µM aptamer at 25 °C. Raw ITC data (top) and enthalpy change plotted against the COC:aptamer molar ratio (down).

Table S4. ITC results of COC and MET aptamers

Aptamer	Sample Cell Concentration	Syringe Concentration	K_d	ΔH kcal/mol	ΔG kcal/mol
COC	20 µM	450 µM	306 nM	-80	-8.90
MET	25µM	250 µM	570 nM	-45.1	-9.30

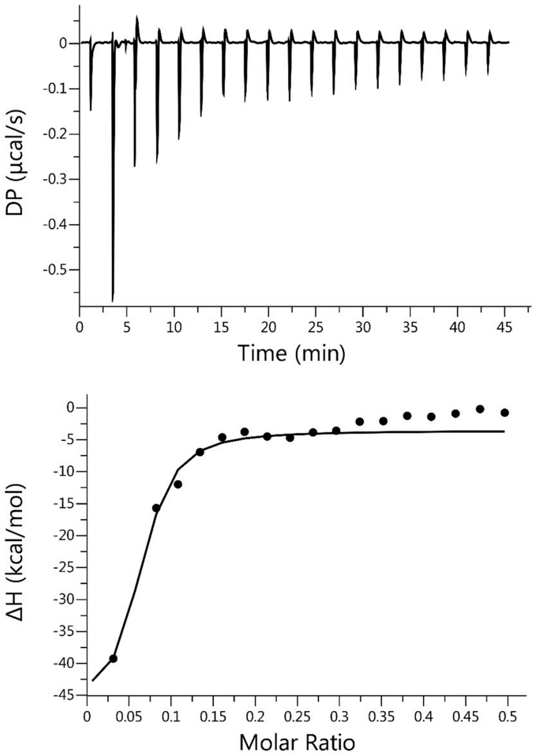

Figure S4. Molecular interactions of MET with MET aptamer measured by ITC. 250 µM MET was titrated into 20 µM aptamer at 25 °C. Raw ITC data (top) and enthalpy change plotted against the MET:aptamer molar ratio (down).

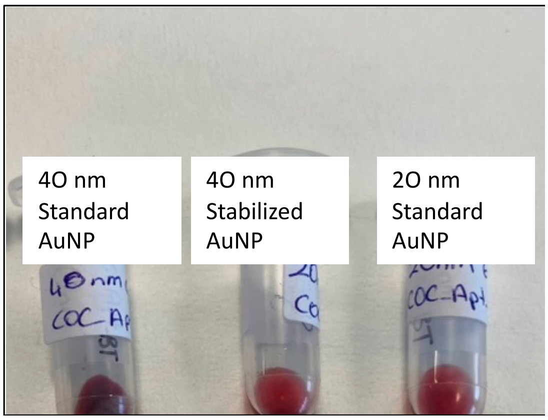

Figure S5. Conjugates prepared with standard and stabilized gold nanoparticles (AuNPs).

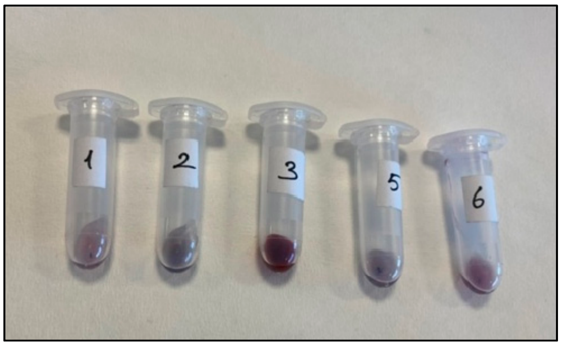

Figure S6. Conjugates blocked under different conditions.
1) Apt/AuNP ratio 1/20 2.0 µL 28 mM MCH 10 min.
2) Apt/AuNP ratio 1/20 2.0 µL 14 mM MCH 10 min.
3) Apt/AuNP ratio 1/20 10 µL 10 mg/mL BSA 30 min.
4) Apt/AuNP ratio 1/20 Unblocked
5) Apt/AuNP ratio 1/10 5.0 µL 10mM MCH 10 min.
6) Apt/AuNP ratio 1/10 5.0 µL 100mM MCH 1 min.

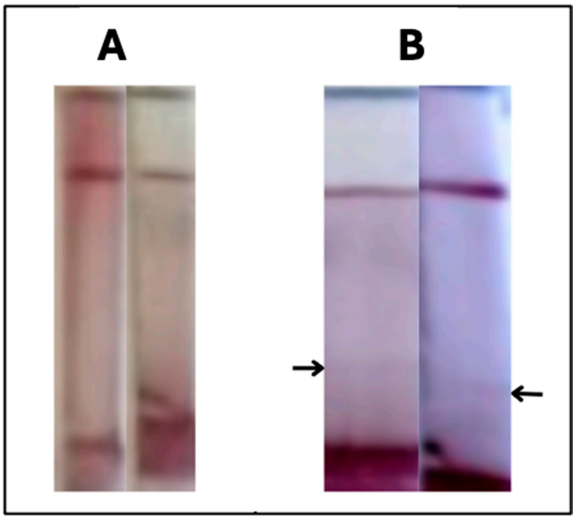

Figure S7. Strip prepared with A.) unblocked conjugate, B.) BSA blocked conjugate.

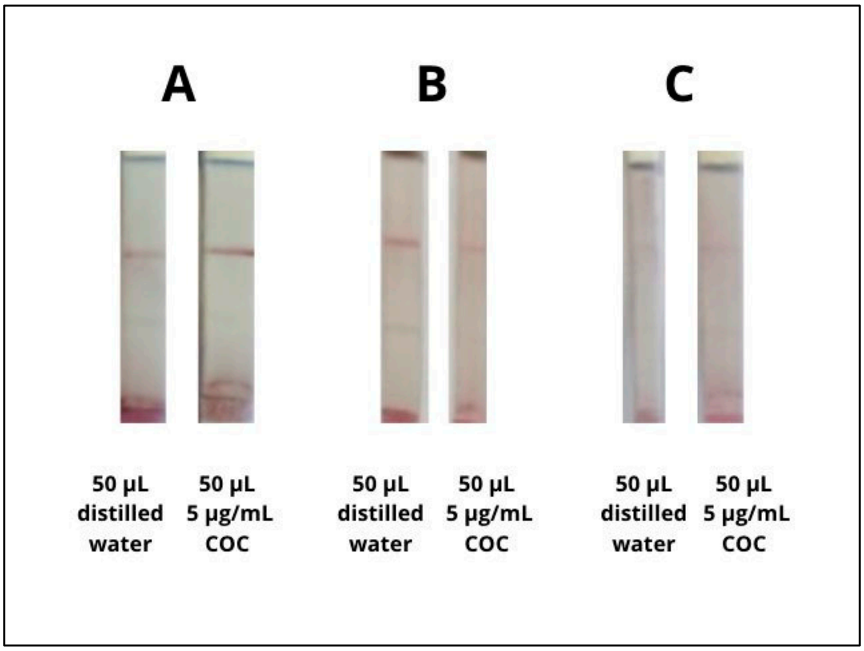

Figure S8. COC strips with A.) 5.0 µM aptamer concentration B.) 10 µM aptamer concentration C.) 25 µM aptamer concentration

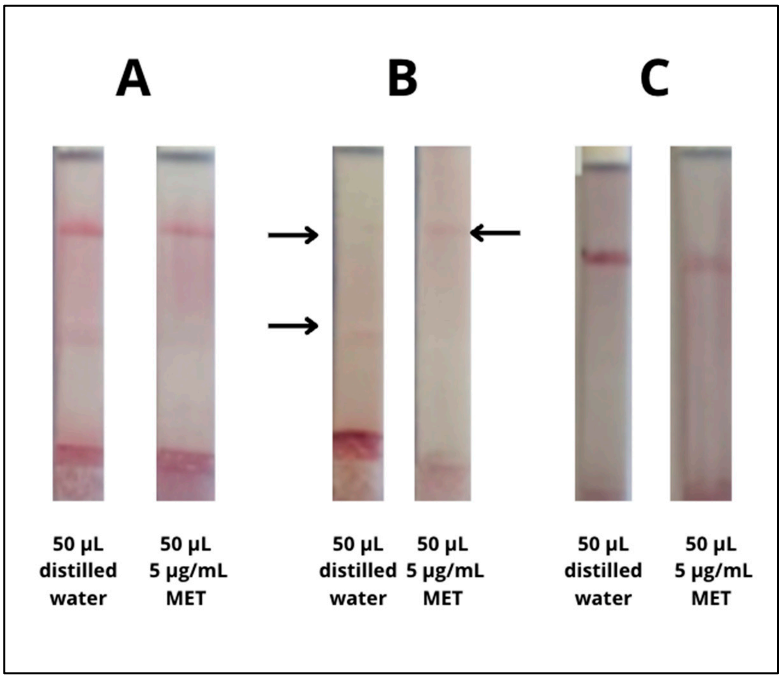

Figure S9. MET strips with A.) 5.0 µM aptamer concentration B.) 10 µM aptamer concentration C.) 25 µM aptamer concentration

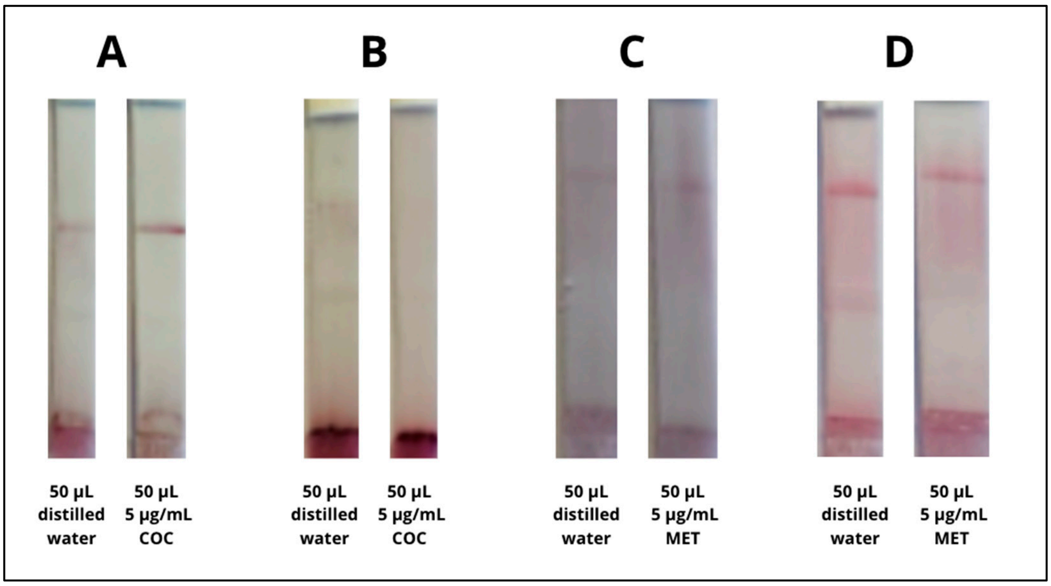

Figure S10. A.) Strip prepared with aptamer without biotin for COC B.) Strip prepared with biotin-tipped aptamer for COC C.) Strip prepared with biotin-tipped aptamer for MET D.) Strip prepared with aptamer without biotin for MET.

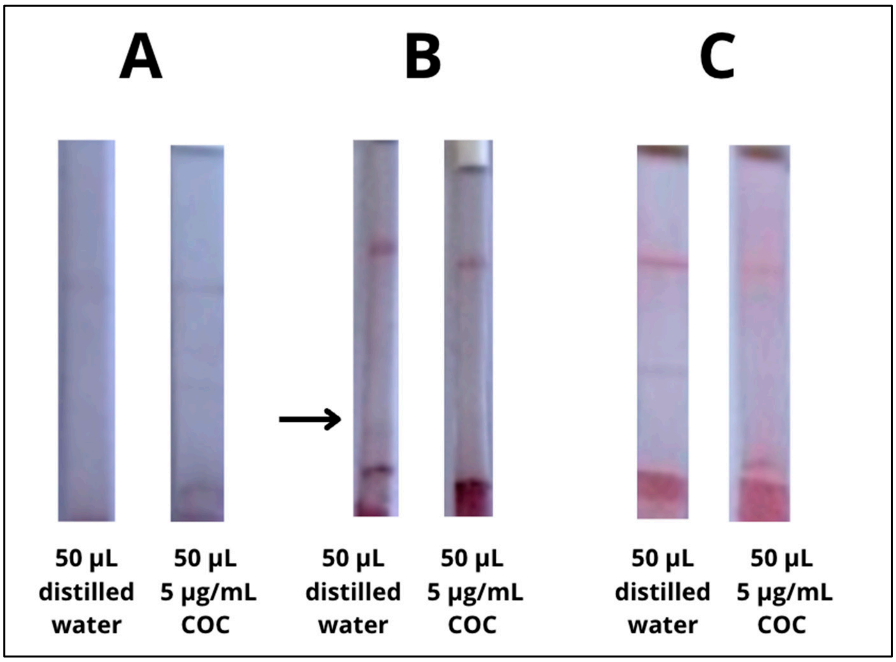

Figure S11. COC Strips A.) Strip prepared with conjugate with final concentration of 30 µM NaCl B.) 70 µM NaCl C.) 100 µM NaCl.

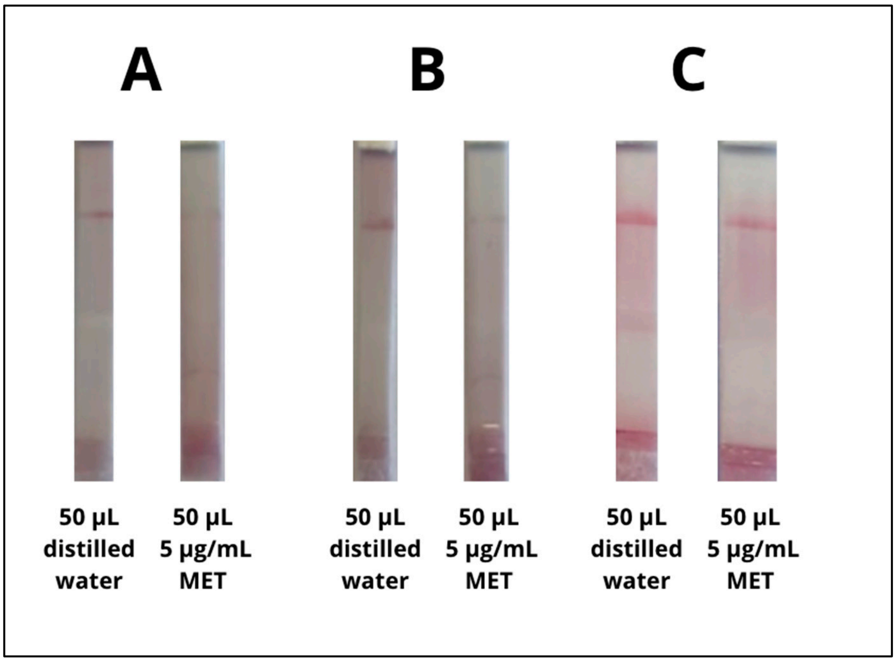

Figure S12. MET Strips A.) Strip prepared with conjugate with final concentration of 30 µM NaCl B.) 70 µM NaCl C.)100 µM NaCl.

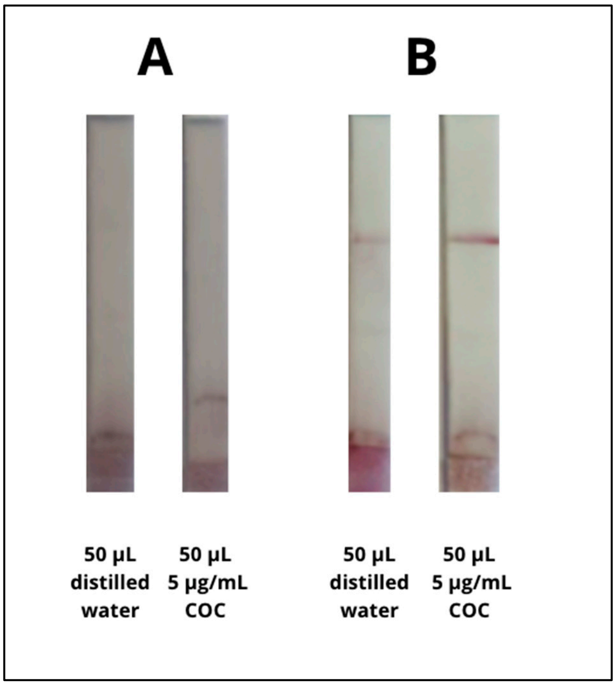

Figure S13. A.) Nitrocellulose membrane with blocking solution B.) Nitrocellulose membrane without blocking solution.

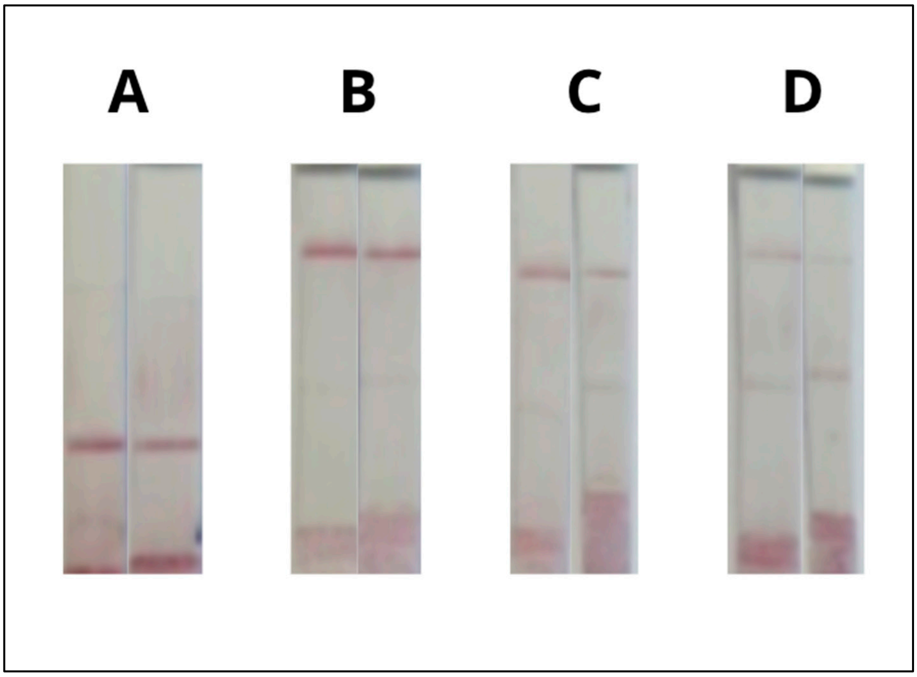

Figure S14. Negative sample applications on strips prepared by dilutions applied to conjugate. A.) 35 µL of undiluted conjugate was impregnated into the conjugate pad. B.)15 µL conjugate was diluted with 60 µL conjugate blocking solution. C.) 25 µL conjugate was diluted with 50 µL conjugate blocking solution. D.) 37.5 µL conjugate was diluted with 37.5 µL conjugate blocking solution.

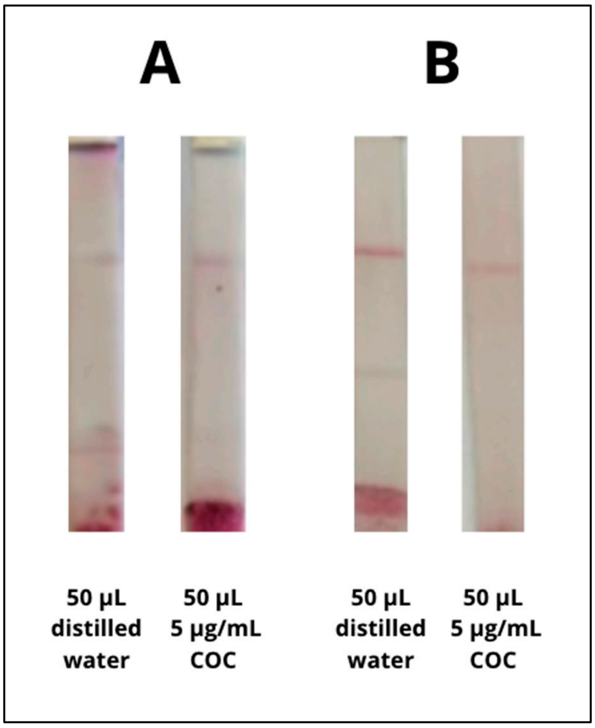

Figure S15. A.) 1.0 mg/mL concentration antigen test strip B.) 2.0 mg/mL concentration antigen test strip.

3.4 Drying Conditions

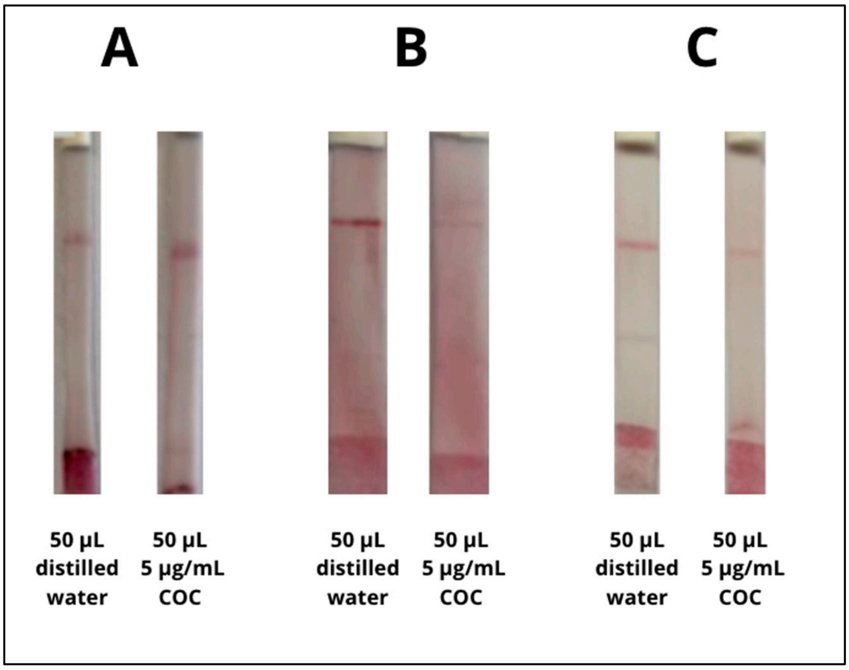

Figure S16. Strip formed by membrane dried at A.) 37 °C for 2 h under 20% moisture B) 37 °C for 2 h under moisture-free conditions C.) 37 °C for 30 min under moisture-free conditions.

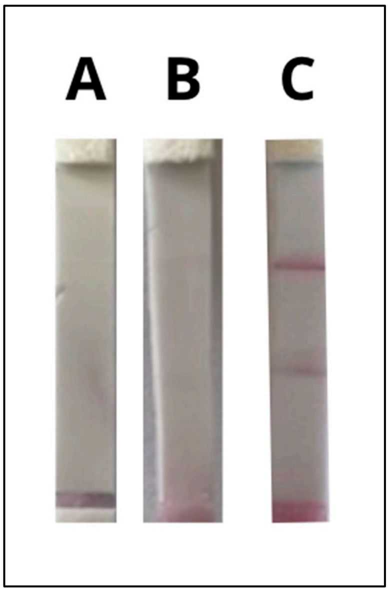

Figure S17. Strip prepared with A) Ahlstorm Grade 238 cotton fibre pad B) Ahlstorm Grade 601 cotton fibre pad C) Ahlstorm glass fiber pad.
